# Mechanical Characterization, Water Absorption, and Thickness Swelling of Lightweight Pineapple Leaf/Ramie Fabric-Reinforced Polypropylene Hybrid Composites

**DOI:** 10.3390/polym16131847

**Published:** 2024-06-28

**Authors:** Lin Feng Ng, Mohd Yazid Yahya, Chandrasekar Muthukumar, Jyotishkumar Parameswaranpillai, Quanjin Ma, Muhammad Rizal Muhammad Asyraf, Rohah Abdul Majid

**Affiliations:** 1 Centre for Advanced Composite Materials (CACM), Universiti Teknologi Malaysia, Johor Bahru 81310, Malaysia; r-rohah@utm.my; 2 Faculty of Mechanical Engineering, Universiti Teknologi Malaysia, Johor Bahru 81310, Malaysia; 3 SIMCRASH Centre, Department of Aerospace Engineering, Hindustan Institute of Technology & Science, Kelambakkam, Chennai 603103, India; chandrasm@hindustanuniv.ac.in; 4 AU—Sophisticated Testing and Instrumentation Center, Alliance University, Chandapura-Anekal Main Road, Bengaluru 562106, India; jyotishkumar.p@alliance.edu.in; 5 School of System Design and Intelligent Manufacturing, Southern University of Science and Technology, Shenzhen 518055, China; maqj@sustech.edu.cn; 6 Department of Bioprocess and Polymer Engineering, Faculty of Chemical and Energy Engineering, Universiti Teknologi Malaysia, Johor Bahru 81310, Malaysia

**Keywords:** hybrid composites, woven fabric, ramie fiber, pineapple leaf fiber, mechanical properties, water absorption, thickness swelling, fiber stacking configuration

## Abstract

Fiber-reinforced composites are among the recognized competing materials in various engineering applications. Ramie and pineapple leaf fibers are fascinating natural fibers due to their remarkable material properties. This research study aims to unveil the viability of hybridizing two kinds of lignocellulosic plant fiber fabrics in polymer composites. In this work, the hybrid composites were prepared with the aid of the hot compression technique. The mechanical, water-absorbing, and thickness swelling properties of ramie and pineapple leaf fiber fabric-reinforced polypropylene hybrid composites were identified. A comparison was made between non-hybrid and hybrid composites to demonstrate the hybridization effect. According to the findings, hybrid composites, particularly those containing ramie fiber as a skin layer, showed a prominent increase in mechanical strength. In comparison with non-hybrid pineapple leaf fabric-reinforced composites, the tensile, flexural, and Charpy impact strengths were enhanced by 52.10%, 18.78%, and 166.60%, respectively, when the outermost pineapple leaf fiber layers were superseded with ramie fabric. However, increasing the pineapple leaf fiber content reduced the water absorption and thickness swelling of the hybrid composites. Undeniably, these findings highlight the potential of hybrid composites to reach a balance in mechanical properties and water absorption while possessing eco-friendly characteristics.

## 1. Introduction

Fiber-reinforced composites are versatile materials with superior specific mechanical properties [1,2]. Towards the goal of zero waste and a sustainable environment, developing high-performance green materials is paramount in battling climate change resulting from anthropogenic pollution. In this regard, lignocellulosic fibers stand out as a viable option for reinforcing composite materials. Growing environmental concerns and stringent government policies have resulted in significant attention being given to lignocellulosic fibers [3,4,5,6]. They possess innumerable attractive characteristics that have a high potential to replace synthetic fibers in the market. Light weight, high specific properties, environmental friendliness, high abundance, low abrasion, inexpensiveness, and low energy consumption during processing are the most commonly cited virtues of lignocellulosic fibers [7,8,9,10,11,12]. Among all the benefits, light weight and high specific properties are promising as they can reduce energy consumption in various engineering sectors without significantly compromising safety performance [13]. Compared with glass fiber, lignocellulosic fibers exhibit a lower density and can be utilized to develop lightweight composites [14,15]. In transportation sectors, weight reduction helps reduce the fuel consumption required to generate energy to drive vehicles. This reduction in fuel consumption may lessen the greenhouse gas emissions from vehicles [16]. Similar to transportation fields, weight reduction is also beneficial to the construction sector as it lessens the energy consumption required to operate heavy machinery and equipment. Moreover, it has been shown that using environmentally friendly materials in the construction sector can reduce the carbon footprint annually and mitigate the pollution level [17]. Due to the environmentally friendly and low-cost characteristics of lignocellulosic fibers, they are preferable over synthetic fibers [18].

The mechanical properties of lignocellulosic fibers are primarily determined by their chemical composition and microfibrillar angles [19]. Generally, fibers with high cellulose content have a higher mechanical strength and stiffness since cellulose is the principal chemical component providing structural support in plants. It is worth noting that higher lignin content could serve as a barrier against moisture absorption due to the hydrophobic characteristics of lignin. From this perspective, it is possible to develop biocomposites with high mechanical properties and great moisture resistance, and this can be realized by merging two different kinds of lignocellulosic fibers with high cellulose and lignin contents, respectively. Among all the lignocellulosic fibers, ramie and pineapple leaf fiber (PALF) manifest remarkable mechanical strength and stiffness, thus showing high potential to be utilized as reinforcements for composite materials. Ramie has been reported to have excellent absolute and specific mechanical properties and resistance to microbial erosion [20,21]. It has been used as a textile material to make garments due to its excellent moisture absorption and breathability [22]. Considering its high mechanical strength and toughness, it provides an opportunity to expand its applications to the automotive industry. On the other hand, PALF is normally abandoned as agricultural waste after the fruit is harvested, but it displays outstanding mechanical strength and stiffness owing to its high cellulose content and low microfibrillar angle [23,24,25,26,27]. Currently, the potential of such fiber to be used as reinforcement has not been fully explored. Therefore, using PALF as a reinforcement material can be an effective way to develop value-added products while mitigating the issue of waste accumulation. Several studies have been carried out to optimize the mechanical properties of ramie fiber- and PALF-reinforced composites through hybridization with synthetic fibers [28,29,30,31]. In order to further reduce the reliance on synthetic fibers, it is vital to explore the potential of natural/natural hybrid composites rather than natural/synthetic hybrid composites.

Numerous investigations have examined the feasibility of combining various fiber types in composite materials through hybridization. Ramasubbu et al. [32] investigated the mechanical and water absorption characteristics of epoxy hybrid composites reinforced with sisal and kenaf fibers. They reported that non-hybrid kenaf fiber-reinforced composites exhibited the highest tensile strength (TS). It is worth noting that sisal/kenaf hybrid composites exhibited greater impact strength (IS) and lower water absorption compared to non-hybrid composites. In a similar work, Bekele et al. [33] investigated the mechanical and water absorption properties of polyester hybrid composites reinforced with enset/sisal fiber. In terms of mechanical properties, the incorporation of sisal fiber into enset/polyseter composites increased TS and flexural strength (FS) while reducing IS. As for water absorption, hybrid composites with an enset/sisal ratio of 50:50 showed the lowest water absorbing capacity. Tezara et al. [34] investigated the mechanical and water absorption properties of woven jute and woven ramie fiber fabric-incorporated epoxy hybrid composites. In accordance with the findings obtained, incorporating ramie fiber in the jute/epoxy composites increased the mechanical properties, but water resistance was reduced. In addition, they noticed a drop in the mechanical properties of the composites with soaking times in water, regardless of the fiber types. Feng et al. [35] investigated the mechanical and water absorption properties of polypropylene (PP) hybrid composites reinforced with kenaf and PALF. They revealed interesting results in which the balance in mechanical properties and water absorption resistance could be attained through hybridizing kenaf fiber and PALF in hybrid composites due to the higher mechanical strength and stiffness of PALF and the greater water resistance of kenaf fiber. Ismail et al. [36] analyzed the mechanical and water absorption properties of woven kenaf/bamboo fiber-reinforced epoxy hybrid composites with varying relative fiber ratios. Among all the hybrid composites, the hybrid composites with a relative fiber ratio of 50:50 (kenaf:bamboo) showed the highest flexural properties. However, the highest flexural properties were noticed in non-hybrid bamboo fiber composites. Surprisingly, hybrid composites with a 50:50 relative fiber ratio had the highest IS, surpassing non-hybrid bamboo and kenaf fiber composites. When scrutinizing the water absorption of composites, the addition of bamboo fiber could drastically limit the ability of the materials to absorb water.

Thus far, scientific research focusing on natural/natural fiber fabric-reinforced hybrid composites is limited. According to the literature, the mechanical properties and water absorption behavior of woven ramie/PALF-reinforced PP hybrid composites remain unexplored. Given the high strength characteristics of both ramie fiber and PALF and the high economic value of PALF, this research study attempts to explore the mechanical properties and water absorption behavior of ramie/PALF hybrid PP composites with varying fabric stacking configurations. This research work is expected to provide a significant contribution to environmental friendliness and research communities.

## 2. Materials and Methods

### 2.1. Materials

**Figure 1:**
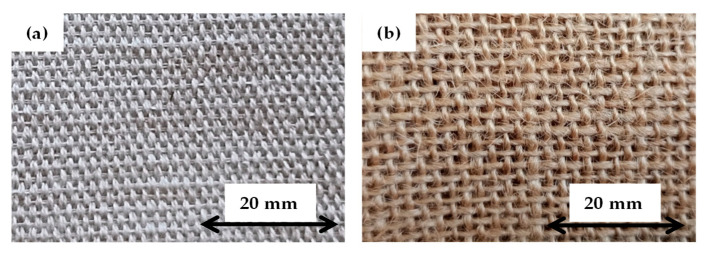
Woven fabrics: (**a**) Ramie, (**b**) PALF.

PT Berkah Rami Neforindo in Central Java, Indonesia supplied the woven ramie fabrics with an areal density of 390 g/m^2^. MechaSolve Engineering in Selangor, Malaysia supplied the woven PALF fabric with an areal density of 315 g/m^2^. The PP granules with a density of 0.91 g/cm^3^ and melt flow index of 12 g/10 min were procured from Al Waha petrochemical company in eastern province, Saudi Arabia. Figure 1 shows the woven fabrics used in this research study. Table 1 summarizes the compiled properties of ramie fiber and PALF.

### 2.2. Sample Preparation

**Figure 2:**
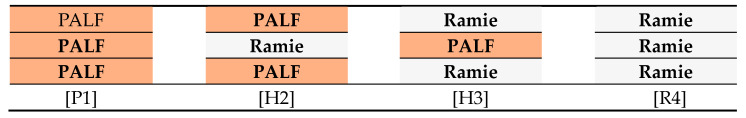
Fabric stacking configurations in PP composite laminates.

The ramie/PALF hybrid PP composites with varying fabric stacking configurations were prepared with the aid of the heat compression approach. The fabrics were stacked alternately with six PP films in a frame mold with dimensions of 250 mm × 250 mm × 3 mm. One PP film was placed, respectively, at the top and bottom of the fabrics, while four other PP films were inserted into the middle fabric interfaces. A total of three fabrics were fixed in the composite laminates regardless of the fabric stacking configurations and fiber types. Prior to the heat compression, the stack was preheated at a temperature of 175 °C for two minutes without applying pressure to allow the heat to be evenly distributed over the composite laminate. The composite laminates were then heat-compressed at the same temperature and pressure of 3 MPa for eight minutes. The composite laminates were then removed from the hot press machine and allowed to cool naturally to room temperature. In this work, non-hybrid and hybrid composite laminates with four fabric stacking configurations were prepared. Figure 2 shows the fabric stacking configurations of the composite laminates. Non-hybrid PALF- and ramie fabric-reinforced composite laminates are, respectively, represented by [P1] and [R4]. Hybrid composite laminates, where the outer layers consist of PALF and the inner core is made of ramie fiber, are denoted as [H2]. [H3] represents hybrid composite laminates with PALF as the core and ramie as the skin layers. Table 2 presents the weight and volume fractions of fibers in both non-hybrid and hybrid composites reinforced with PALF and ramie fibers.

### 2.3. Experimental Methods

Mechanical, water absorption, and thickness swelling tests were conducted to identify the effects of hybridization and fabric stacking configurations on the material properties. The tensile tests were performed at ambient temperature according to ASTM D3039 using an Instron 5982 Universal Testing Machine to determine the TS and tensile modulus (TM). The cross-head displacement rate was fixed at 2 mm/min for all tensile test samples. An extensometer was utilized to monitor the strain of the composite laminates during the tensile tests. The flexural tests were performed at room temperature according to ASTM D790 using the same Instron machine. The span-to-depth ratio of the composite laminates was set at 16:1, and the cross-head displacement rate was fixed at 2 mm/min during the flexural tests. Charpy impact tests were carried out with reference to ASTM D6110 using a ZwickRoell impact tester to determine the energy-absorbing capacity of the composite laminates with varying fabric stacking configurations.

The water absorption tests were conducted in accordance with ASTM D570 to investigate the water-absorbing behaviors of PP composite laminates based on PALF/ramie fibers. The samples were immersed in distilled water until fully saturated over a specified duration. Before conducting the water absorption tests, the initial weight of the dry samples was measured and documented. In order to evaluate the percentage of water absorption, the composite samples were removed from the distilled water at predetermined intervals. The wet surfaces of the specimens were cleaned to eliminate excess water, and the weight of the wet specimens after water absorption was determined. These procedures were repeated until the saturation point was attained. The mass difference between the initial weight and weight after water uptake at each time interval was required to identify the water absorption percentage in accordance with Equation (1) [39]. Subsequently, a graph of the water uptake percentage as a function of the square root of time was then plotted.

(1) $$Water\;absorption\;\left(\%\right)=\frac{M_{t}-M_{0}}{M_{0}}\times 100$$

Here, $M_{t}$ is the weight of the specimens after water absorption at certain time intervals and $M_{0}$ is the weight of the dry specimens before immersion in distilled water.

The rate of water diffusion into the composite laminates was also ascertained by calculating the diffusion coefficient, *D*, of the water absorption. Equation (2) was utilized to identify the diffusion coefficient of each composite laminate by considering the slope of the water uptake percentage–square root of the time graph [39].

(2) $$Diffusion\;coefficient,\;D=\pi {\left(\frac{h}{4M_{\infty }}\right)}^{2}{\left(\frac{M_{2}-M_{1}}{\sqrt{t_{2}}-\sqrt{t_{1}}}\right)}^{2}$$

Here, h is the average composite thickness, $M_{\infty }$ is the water absorption percentage at the saturation point, $M_{2}-M_{1}$ is the difference between the water absorption percentage at the linear region, and $\sqrt{t_{2}}-\sqrt{t_{1}}$ is the difference of the square root of time at the linear region.

Meanwhile, the sorption coefficient, *S*, of the composite laminates was investigated to indicate their water diffusion resistance. The sorption coefficient was calculated according to Equation (3) [40].

(3) $$Sorption\;coefficient,\;S=\frac{M_{\infty }}{M_{t}}$$

Here, $M_{t}$ is the water absorption percentage of the composite laminates at a specific time.

Finally, the permeability coefficient, *P*, of the composite laminates, which considers both effects of diffusion and sorption coefficients, was identified based on Equation (4) [40].

(4) Permeability coefficient, P=D×S

The thickness swelling tests were performed by measuring the thickness of the composite laminates using a Vernier caliper at certain time intervals during the water absorption test. The thicknesses of the composite laminates before and after the water absorption were determined to calculate the thickness swelling. The thickness swelling of the composite laminates was identified using Equation (5) [41].

(5) $$Thickness\;swelling\;\left(\%\right)=\frac{T_{t}-T_{0}}{T_{0}}\times 100$$

Here, $T_{t}$ is the thickness of the composite specimens after water absorption at certain time intervals and $T_{0}$ is the thickness of the dry specimens before immersion in distilled water.

The morphological analysis on both the non-hybrid and hybrid PALF/ramie fiber fabric-reinforced composites was carried out using a JSM-6010 PLUS/LA scanning electron microscope (SEM). After the tensile tests, the fracture behaviors and the fiber–matrix interfaces of each composite laminate were revealed.

## 3. Results and Discussion

### 3.1. Tensile Properties

The tensile and flexural properties of the PALF/ramie fabric-reinforced PP composites are summarized in Table 3. The findings demonstrated that the hybridization of PALF and ramie fiber resulted in a positive hybrid effect, which significantly improved the tensile properties of the composite laminates. Table 3 shows that increasing the ramie fiber content improves the TS of the hybrid composite laminates. The higher strength of ramie fabric than PALF contributes to a greater TS of the hybrid composite laminates. When comparing [R4] non-hybrid composite laminates to other composite laminates, the TS was the highest due to the superior strength of ramie fabric. Conversely, the TS was found to be the lowest in the non-hybrid [P1] composite laminates. When compared to non-hybrid [P1] composite laminates, [R4] composite laminates have a TS that is 103.65% higher than [P1] composite laminates. This indicates the tremendous potential of ramie fiber in enhancing the mechanical strength of the composites. Due to the high strength characteristic of ramie fabric, incorporating such fiber in the composite laminates was found to enhance the mechanical strength of hybrid composites. By substituting the middle PALF with the ramie fiber layer in [H2] PP composite, the TS was increased by 6.58%. Interestingly, a sharp increase in the TS was noticed when replacing the outer PALF with ramie fiber layers in [H3] composite laminates. [H3] composite laminates have a TS that is 52.10% higher than [P1] composite laminates. Placing high-strength fabric as the skin layers might greatly increase the strength of the composite laminates since the outermost fiber layers are essential for sustaining the tensile stress. These findings are in agreement with the previous literature, in which hybrid composite laminates with high-strength fiber as the skin layers had greater mechanical strength [34,42,43]. Apart from the fiber strength, fabric density also has a significant impact on the composite strength as more yarns may help sustain the external load when the fabric density is high. As mentioned by Patti et al. [44], the TS of the fabric can be improved by reducing the yarn–yarn spacing and increasing the yarn size. As visible in Figure 1, the fabric density of ramie fiber is apparently higher than PALF, resulting in the higher fabric strength of ramie fiber.

**Figure 3:**
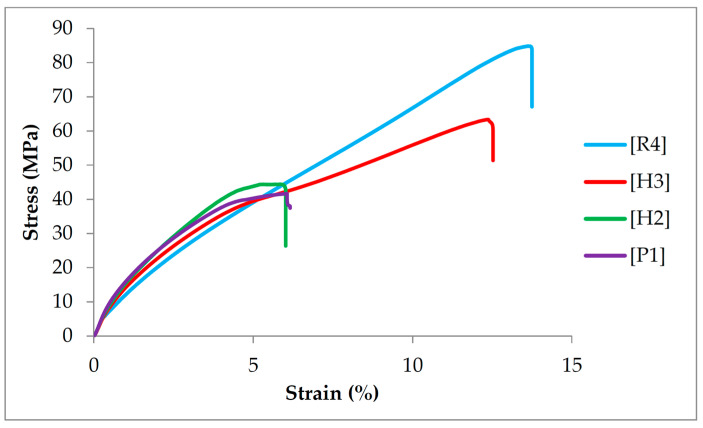
Stress–strain curves of PALF/ramie fiber fabric-reinforced composites.

When the TM of the PALF/ramie fabric-reinforced PP composite laminates was investigated, a different trend emerged, with the TM increasing with an increase in the PALF percentage. The [P1] non-hybrid composite laminates had the highest TM, whereas the [R4] non-hybrid composite laminates had the lowest. When their tensile moduli are compared, [P1] composite laminates have a TM that is 50.39% higher than [R4] composite laminates. When the middle ramie in [H3] composite laminates was replaced with a PALF layer, there was a 24.03% improvement. A further increment in the TM up to 37.98% was achieved by superseding the outermost ramie fiber with PALF layers in [H2] composite laminates. The higher TM of the PALF-based composite laminates can be attributed to the better mechanical interlocking of the PALF woven structure. When the composite laminates experienced a tensile load, the mechanical interlocking of warp and weft yarns in PALF fabric resisted the deformation, thus offering a higher TM. When referring to Figure 1, the waviness of PALF fabric is greater than that of ramie fabric due to the higher linear density of weft and warp yarns of PALF fabrics. In other words, PALF fabrics consist of coarser fiber yarns than ramie fabrics. Furthermore, the PALF yarns in the warp and weft directions encompass similar linear densities. Unlike the PALF fabric, the linear density of the ramie weft yarn is prominently smaller than that of warp yarn, leading to lower waviness and mechanical interlocking due to a lesser contact angle. However, the waviness of the PALF layer may result in deterioration in mechanical strength due to a large sliding angle which may induce higher shear stress at the yarn interfaces. Asgarinia et al. [45] reported that high crimp levels promoted inordinate stress at the yarn interfaces, leading to transverse and interfacial cracks. Figure 3 shows the stress–strain plots of PALF/ramie fabric-reinforced composites with varying fabric stacking configurations. All the curves show an increment in stress with an increase in strain, regardless of fiber type and fabric stacking configuration. Nonetheless, the stress–strain curves of [P1] and [H2] composite laminates show similar behaviors, whereas the stress–strain curves of [H3] and [R4] composite laminates are comparable, implying that the tensile properties of composite laminates are heavily influenced by the outermost skin layers. In other words, the outermost fiber layers are responsible for the tensile properties of PP composites.

### 3.2. Flexural Properties

Flexural properties, such as the FS and flexural modulus (FM) of PALF/ramie fiber-reinforced PP composites with different fabric stacking configurations, were determined. The flexural properties of the composite laminates are recorded in Table 3. The FS showed a similar trend to that of the TS of the composite laminates. According to Table 3, non-hybrid [R4] composite laminates exhibit the highest FS, while [P1] composite laminates demonstrate the lowest FS. The FS of [R4] composite laminates was found to be 88.30 MPa, which is 39.60% higher than that of [P1] composite laminates. Despite the lowest FS of [P1] composite laminates, improvement can be achieved by substituting middle PALF with ramie fabric in [H2] composite laminates. With the partial replacement of the middle PALF with ramie fabric, the FS was increased by 5.55%. A more prominent improvement of up to 52.10% was obtained when superseding the outermost PALF layers with ramie fabrics in [H3] composite laminates. In comparison with PALF, the higher compactness of ramie fiber, as shown in Figure 1, allowed more fiber yarns to sustain the flexural load, thereby enhancing the FS of the composite laminates.

**Figure 4:**
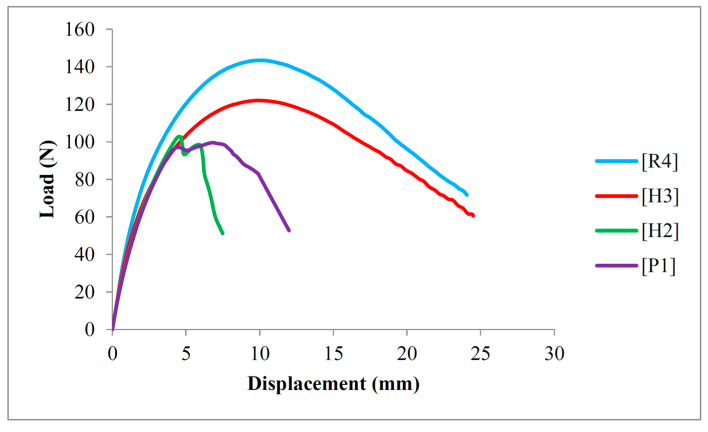
Load-displacement curves of PALF/ramie fabric-reinforced composites.

Unlike the TM, the highest FM was found in [R4] composite laminates, whereas the lowest FM was identified in [P1] composite laminates. These results suggest that an increased proportion of ramie content contributed to a rise in the FM of composite laminates. Specifically, the FM of [R4] composite laminates was measured at 3.73 GPa, representing a 29.97% increase compared to [P1] composite laminates. As for the hybrid composite laminates, [H2] and [H3] displayed FMs of 2.91 GPa and 3.25 GPa, which are 1.39% and 13.24%, respectively, higher than [P1] composite laminates. These results indicated that the interlacement of warp and weft yarns in PALF fabric did not significantly influence the FM of the composite laminates. This is most likely due to the fact that, unlike the tensile test, which subjected the entire specimen to a uniaxial load, only a small section of the specimen was subjected to a bending load, resulting in a minimal effect of mechanical interlocking between the warp and weft yarns of PALF. Furthermore, the high compactness of the ramie fabric, as shown in Figure 1, allowed more fiber yarns to sustain the flexural load, thus enhancing the FM of the composite laminates. Figure 4 displays the load-displacement curves of the PALF/ramie fabric-reinforced composites with various fabric stacking configurations. Upon examining the load-displacement curves of the composite laminates, it is evident that the flexural properties of the materials are significantly influenced by the outermost layers of skin. The top and bottom fabric layers withstood the bending load, whereas the core component distributed the load uniformly within the composite laminates. This is evidenced by the similar flexural behaviors of [P1] and [H2] composite laminates. Moreover, the flexural behaviors of [H3] and [R4] composite laminates were very similar. During the flexural tests, the top skin layer was subjected to compression, while the bottom skin layer was subjected to tension, indicating that the outermost fabric layers were critical in resisting the flexural load. As the flexural load is mainly sustained by the outermost layers of composite laminates, placing high-strength fabrics as the skin layers is considered more judicious in developing advanced composite materials.

### 3.3. Impact Properties

**Figure 5:**
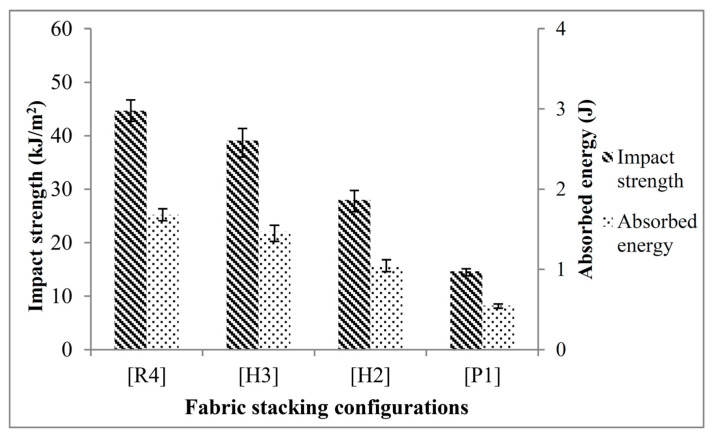
Impact strength of PALF/ramie fabric-reinforced PP composites.

The impact properties of the PP composites with distinct fabric stacking configurations were assessed to gauge their ability to absorb energy. The impact properties of the PP composite laminates are known to be significantly impacted by fiber stacking configurations and hybridization. Therefore, this work intends to quantitatively unveil the effect of hybridizing PALF and ramie fabric on the IS of the composite laminates. The absorbed energy and IS of the composite laminates with varying fabric stacking configurations are shown in Figure 5. Analogous to tensile strength and FS, [R4] composite laminates displayed the highest absorbed energy and IS, showing that ramie fabric helped composite laminates have the highest energy-absorbing capacity. In contrast, the lowest absorbed energy and IS were found in [P1] composite laminates. Figure 5 shows that [R4] composite laminates have an IS of 44.67 kJ/m^2^, which is 204.50% higher than [P1] composite laminates. This significant difference between the IS of [R4] and [P1] composite laminates highlights the superior energy-absorbing capacity of ramie fabric. Nevertheless, the impact resistance of the composite laminates increased by applying hybridization, demonstrating a favorable hybrid effect. In accordance with the findings obtained, improvement was observed when hybridizing ramie with PALF in PP composites. It was found that the IS of [H2] composite laminates increased by 90.87% when the center PALF was replaced with ramie fabric. A further enhancement of up to 166.60% was obtained in [H3] composite laminates when substituting the outermost PALF with ramie fabrics. The results indeed demonstrated that hybridizing ramie with PALF could uplift the impact strength of the hybrid composite laminates, which could be beneficial to those impact-critical applications where energy absorption is particularly vital. It has been identified that the impact behaviors of composite materials are more complex than those of metal alloys since they consist of multiple solid phases with distinctive material properties. Aside from fiber type and fabric stacking configuration, the impact energy is absorbed and dissipated through several fracture modes, including fiber breakage, fiber pull-out, and fiber–matrix debonding. Therefore, it is crucial to analyze and evaluate the fracture behaviors of the non-hybrid and hybrid composite laminates to gain a deeper understanding of their impact properties. The fracture behaviors of each composite laminate are shown and discussed in Section 3.6.

### 3.4. Water Absorption Behavior

**Figure 6:**
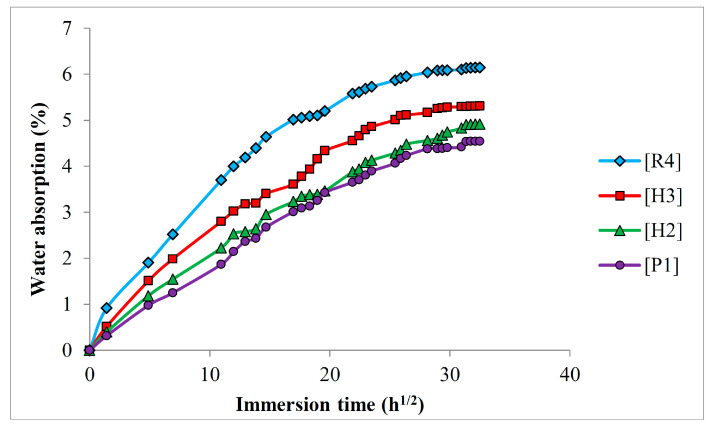
Water absorption plots of PALF/ramie fabric-reinforced PP composites.

The water absorption characteristics in terms of the water absorption percentage, diffusion coefficient, sorption coefficient, and permeability coefficient of PP composites with varying fabric stacking configurations were investigated. It is widely understood that cellulosic natural fibers possess a strong attraction to water molecules, primarily due to the hydrogen bond formation between the hydroxyl groups of the fiber fabrics and water molecules. This characteristic suggests that they have a tendency to absorb substantial quantities of water from their environment. However, the water absorption of natural fibers decreases when they are embedded in hydrophobic polymer matrices. It should be noted that the water absorption of natural fibers is highly dependent on their chemical composition. Thus, hybridization is expected to lessen the water-absorbing capacity and water sensitivity of the composites due to the distinctive chemical compositions of the two different fibers. Figure 6 shows the water absorption characteristics of PALF/ramie fabric-reinforced PP composites with varying fabric stacking configurations. The water absorption plots indicate the water uptake percentages of each non-hybrid and hybrid composite with respect to the square root of the immersion time. All the plots demonstrate a similar pattern where the water uptake percentage increased with an increase in the square root of the immersion time. The plots showed a rapid water uptake at the initial stage of water absorption. After a certain period of immersion time, the water uptake slowed down and eventually reached a saturation point where the water uptake percentage became constant. Similar water absorption behavior of composite materials was also reported in the previous literature [42,46,47].

By referring to Figure 6, it is visible that the water uptake percentage increases with an increase in the ramie fiber content. The highest water uptake percentage was noticed in the non-hybrid ramie fabric-reinforced PP composites, [R4], whereas the non-hybrid pineapple leaf fiber-reinforced composites [P1] recorded the lowest moisture uptake percentage. As expected, the water absorption curves of hybrid composites lie between those of non-hybrid composites. More specifically, incorporating PALF in the hybrid composites reduced their water absorption. Since both PALF and ramie fabrics are cellulosic natural fibers, it is necessary to look into their chemical compositions. Generally, the water absorbing behavior of natural fibers is governed by hemicellulose and lignin contents. Hemicellulose is the main chemical component of natural fiber accountable for water absorption, while the intrinsic hydrophobic behavior of lignin prevents the water from penetrating into the fibers [48,49,50,51]. Hence, natural fibers with high hemicellulose content generally have greater water sensitivity, while those with high lignin content may have better resistance against water absorption. When referring to Table 1, it is obvious that PALF has a relatively low hemicellulose content, implying that the water absorption of PALF is lower than ramie fiber. Aside from lower hemicellulose content, PALF also has a higher lignin content than ramie fiber, allowing such fiber to have greater resistance against water absorption as the lignin content may serve as a protective layer which retards the water from penetrating into the fiber.

Table 4 summarizes the water absorption properties of PALF/ramie fabric-reinforced PP composites. The moisture uptake percentage at saturation point, diffusion coefficient, sorption coefficient, and permeability coefficient are all shown in Table 4. The diffusion coefficient of each composite laminate serves as a measure to assess the ability of water molecules to diffuse into the laminates. Thus, it serves as an indication of how easily the water can diffuse into the composite laminates. From Table 4, [R4] presents the highest diffusion coefficient, followed by [H3] and [H2] hybrid composites. The lowest diffusion coefficient was recorded in [P1] composite laminates, indicating that water molecules were more difficult to diffuse into PALF-incorporated composite laminates. The sorption coefficient, on the other hand, indicates the water resistance of each composite laminate. The sorption coefficient of composite laminates is consistent with their diffusion coefficient, with [P1] composite laminates exhibiting the strongest water resistance and [R4] exhibiting the lowest resistance to water diffusion. Undeniably, increasing PALF content in the hybrid composite laminates improved their water resistance, mitigating the ability of the water molecules to diffuse into the composite laminates. After identifying the diffusion and sorption coefficients, it is essential to determine the permeability coefficient to show the net effect of water absorption. When scrutinizing the permeability coefficient of PALF/ramie fabric-reinforced PP composites, the permeability coefficient of composite laminates is in line with their diffusion coefficient in which [R4] composite laminates showed the highest permeability coefficient while the lowest was found in [P1] composite laminates. The permeability coefficient of hybrid composites, [H2] and [H3], was intermediate of the non-hybrid composites. These findings further prove that PALF has better water resistance owing to its higher lignin content. With the remarkable strength of ramie-incorporated composites and the lower water sensitivity of PALF, it can be expected that a balance in mechanical properties and water absorption can be achieved by applying the hybridization concept.

### 3.5. Thickness Swelling

**Figure 7:**
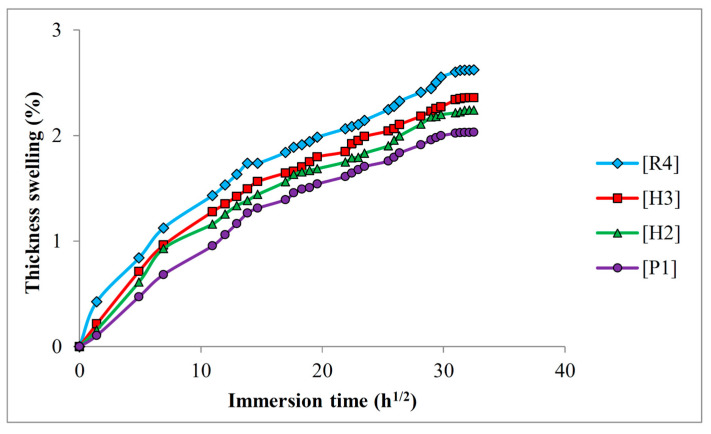
Thickness swelling characteristics of PALF/ramie fabric-reinforced PP composites.

Water absorption may trigger the thickness swelling of composite materials, particularly when it involves hydrophilic plant fibers. From the theoretical point of view, the swelling of cellulosic fibers promotes stress at the interfacial regions, leading to micro-cracking which can intensify the water absorption in the composites [34]. Additionally, swollen fibers can also deteriorate the fiber–matrix adhesion, thereby increasing the water absorption percentage. Water absorption is, therefore, considered a self-accelerated mechanism. Since dimensional stability is vital to ensure optimum material properties, it is necessary to analyze the thickness swelling of non-hybrid and hybrid PALF/ramie fabric-reinforced PP composites. Figure 7 shows the thickness swelling of non-hybrid and hybrid PALF/ramie fabric-reinforced PP composites. The findings from thickness swelling exhibited a trend akin to the water absorption percentages observed in both non-hybrid and hybrid PALF/ramie fabric-reinforced composites. Non-hybrid [R4] composite laminates had the greatest thickness swelling, while non-hybrid [P1] composite laminates had the least thickness swelling. The hybrid composite laminates [H2] and [H3] had thickness swellings that fell between those of the non-hybrid composite laminates. At the end of the water absorption test, the greatest thickness swelling of composite laminates was 2.62% ([R4]), followed by 2.36% ([H3]), 2.24% ([H2]), and 2.03% ([P1]).

Overall, an increase in PALF content reduced the thickness swelling of composite laminates. As mentioned in the previous section, PALF has relatively lower hemicellulose and higher lignin content, eventually resulting in lower water absorption. Due to the lower water absorption of PALF-based composite laminates, their thickness swellings were lower than ramie fiber-based composite laminates. From the perspective of fiber–matrix adhesion, a weak fiber–matrix adhesion could lead to greater water absorption and thickness swelling as the water could be entrapped at the fiber–matrix interfacial region. Kamaruddin et al. [52] reported that a weak fiber–matrix adhesion resulted in a large discrepancy at the interfacial region, allowing rapid water penetration into the composites and leading to high-thickness swelling. In this regard, ramie fiber had a weaker fiber–matrix adhesion level than PALF; thus, more water could be entrapped at the fiber–matrix interfacial region of ramie fiber-based composite laminates, leading to a higher level of thickness swelling. The weak fiber–matrix adhesion of ramie fiber-based composite laminates is shown in the SEM images in the subsequent section to support the justification. From Figure 7, it is clear that the thickness swelling of composite laminates shows a similar trend, irrespective of fiber stacking configurations, despite their different thickness swelling values. All the thickness swelling curves demonstrated an increase in thickness swelling with respect to immersion time. At the initial stage of water absorption, the thickness swelling increased rapidly and became slower when the immersion time increased, eventually reaching a stable and constant thickness swelling at the end of the water absorption test.

### 3.6. Morphological Behavior

**Figure 8:**
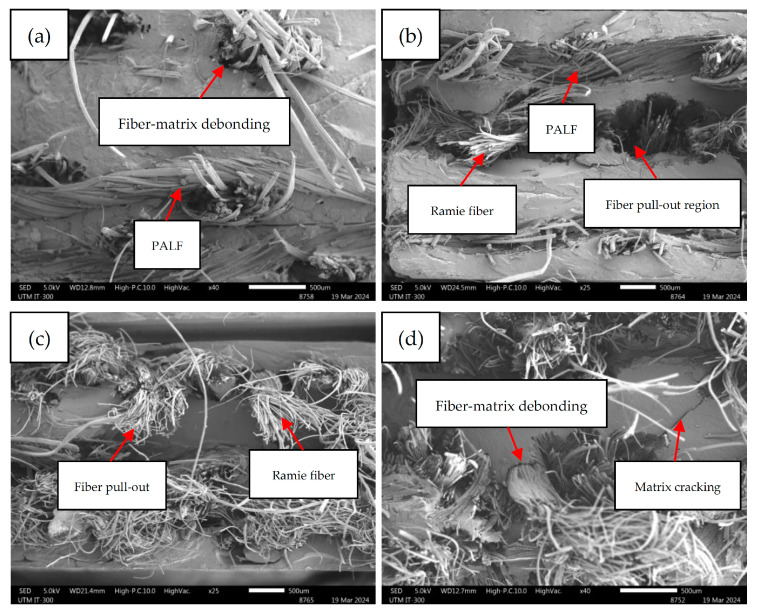
SEM images of non-hybrid and hybrid PALF/ramie fabric-reinforced PP composites: (**a**) [P1], (**b**) [H2], (**c**) [H3], (**d**) [R4].

The tensile fracture surfaces of the non-hybrid and hybrid PP composites were analyzed using SEM. Figure 8 shows the SEM images of each pineapple/ramie fiber fabric-reinforced composite laminate. Notably, the fracture surfaces of the composites were dominated by several fracture behaviors, including fiber pull-out, fiber–matrix debonding, and fiber breakage. These fiber fracture characteristics demonstrated that fibers undoubtedly played a crucial role in resisting the tensile force, thereby uplifting the mechanical strength of the composite laminates. However, when comparing the fracture behaviors of PALF-based and ramie-based composite laminates, it can be observed that fiber pull-out was more significant in ramie fabric-reinforced composites, as shown in Figure 8c,d, indicating that fiber–matrix bonding of PALF-based composites was greater than that of ramie-based composites. Even though fiber–matrix debonding and fiber pull-out might deteriorate the load-transferring efficiency, they could enhance energy dissipation, thus improving the IS of the composite laminates. The woven fabrics were formed by yarn interlacement in warp and weft directions, and each yarn was developed by twisting several strands of fiber. After fiber breakage, yarn untwisting and loosening occurred regardless of fiber type, eventually resulting in fiber splitting, which is especially prominent in SEM images of ramie fiber-based composites.

## 4. Conclusions

The mechanical and water absorption properties of PALF/ramie fabric-reinforced PP composites with varying fabric stacking configurations were investigated in this research study. According to the findings obtained, improvement in the mechanical properties was realized through hybridization. By hybridizing ramie fiber with PALF, a balance in mechanical strength and stiffness can be attained. The tensile, flexural, and Charpy impact strengths showed improvements of 6.58%, 5.55%, and 90.87%, respectively, when one middle PALF layer was replaced with ramie fiber in [H2] composite laminates. Further enhancements in the tensile, flexural, and Charpy impact strengths, up to 52.10%, 18.78%, and 166.60%, respectively, were observed upon substituting the outermost PALF layers with ramie fiber in [H3] composite laminates. Overall, non-hybrid ramie fiber-composite laminates, [R4], displayed the highest mechanical strength. Despite the highest mechanical strength of [R4] composite laminates, [P1] composite laminates presented a higher TM owing to a stronger mechanical interlocking of warp and weft yarns in PALF fabric, providing a greater resistance against deformation. In comparison with TM, the FM of PALF/ramie fabric-reinforced composites showed a different trend in which the [R4] composite laminates manifested the highest FM. This is ascribed to the insignificant effect of mechanical interlocking of PALF under an out-of-plane load. In view of water absorption, incorporating PALF could reduce the water absorption percentage at the saturation point, diffusion coefficient, and permeability coefficient. On the other hand, the sorption coefficient increased with an increase in PALF content. These findings indicate the advantage of using PALF in lowering the water absorption of the hybrid PP composite laminates due to its greater lignin content, which serves as a barrier against water absorption. When looking into the thickness swelling of PALF/ramie fabric-reinforced PP composites, a similar trend to that of the water absorption percentage was obtained. The non-hybrid [R4] composite laminates exhibited the greatest thickness swelling, while the lowest was observed in [P1] composite laminates. The thickness swelling of hybrid composites was in between those of non-hybrid composite laminates. Undeniably, an increase in the PALF content decreased the thickness swelling of the composite laminates. In an effort to mitigate agricultural waste issues, adopting PALF as a reinforcement for composite materials could be a viable option due to its lower water absorption. Towards a sustainable environment, applying the hybridization concept by merging ramie and PALF to develop hybrid composites is expected to attain a balance in mechanical properties and water absorption while preserving the environment. The balance in mechanical properties and water absorption opens an opportunity to employ PALF/ramie fabric-reinforced PP composites in the automotive industry for interior car door panels, dashboards, and package trays.

## Figures and Tables

**Table 1:** Properties of ramie fiber and PALF (data from [37,38]).

Properties	Ramie	PALF
Density (g/cm^3^)	1.0–1.55	0.8–1.6
TS (MPa)	400–1000	180–1627
Elastic modulus (GPa)	24.5–128	1.44–82.5
Cellulose (%)	68.6–85.0	81.27
Hemicellulose (%)	13.0–16.7	12.31
Lignin (%)	0.5–0.7	3.46

**Table 2:** Fiber weight and volume fractions of PP composite laminates.

Fiber Layup	Fiber Weight Fraction (%)	Fiber Volume Fraction (%)		
		PALF	Ramie	Total
[P1]	28.91 ± 0.76	24.30 ± 0.63	–	24.30 ± 0.63
[H2]	29.60 ± 0.75	23.45 ± 0.48	8.68 ± 0.28	32.13 ± 0.76
[H3]	32.36 ± 1.35	8.04 ± 0.01	18.73 ±0.03	26.77 ± 0.04
[R4]	34.56 ± 0.90	–	28.77 ± 0.38	28.77 ± 0.38

**Table 3:** Tensile and flexural properties of PALF/ramie woven fabric-reinforced PP composites.

Fabric Stacking Configuration	Tensile Properties		Flexural Properties	
	TS (MPa)	TM (GPa)	FS (MPa)	TM (GPa)
[R4]	84.82 ± 1.72	1.29 ± 0.25	88.30 ± 1.39	3.73 ± 0.32
[H3]	63.35 ± 1.19	1.60 ± 0.16	75.13 ± 1.44	3.25 ± 0.24
[H2]	44.39 ± 1.94	1.78 ± 0.15	66.76 ± 0.31	2.91 ± 0.17
[P1]	41.65 ± 2.27	1.94 ± 0.18	63.25 ± 2.74	2.87 ± 0.34

**Table 4:** Water absorption characteristics of PALF/ramie fabric-reinforced PP composites.

Fabric Stacking Configuration	Water Uptake Percentage at Saturation Point (%)	Diffusion Coefficient, D × 10^−8^ (m^2^/s)	Sorption Coefficient	Permeability Coefficient, P × 10^−8^ (m^2^/s)
[R4]	6.14	1.97	3.21	6.32
[H3]	5.31	0.84	3.52	2.96
[H2]	4.92	0.57	4.13	2.35
[P1]	4.54	0.43	4.63	1.99

## Data Availability

The datasets presented in this article are not readily available because the data are part of an ongoing study.
